# Perceived neighborhood safety and firearm secure storage: findings from the 2024 behavioral risk factor surveillance system

**DOI:** 10.1186/s40621-026-00657-6

**Published:** 2026-01-30

**Authors:** Alexander Testa, Karyn Fu, Muhammed Ahmed, Daniel C. Semenza, Dylan B. Jackson, Sandra McKay, Kyle T. Ganson, Jason M. Nagata

**Affiliations:** 1 Department of Management, Policy and Community Health, School of Public Health, University of Texas Health Science Center at Houston, Houston, TX USA; 2 Rice University, Houston, TX USA; 3 University of Houston, Houston, TX USA; 4 Department of Sociology, Anthropology, and Criminal Justice, Rutgers University, Camden, NJ USA; 5 Department of Urban-Global Public Health, School of Public Health, Rutgers University, Piscataway, NJ USA; 6 New Jersey Gun Violence Research Center, Rutgers University, Piscataway, NJ USA; 7 Johns Hopkins Bloomberg School of Public Health, Baltimore, MD USA; 8 Department of Pediatrics, McGovern Medical School, University of Texas Health Science Center at Houston, Houston, TX USA; 9 Factor-Inwentash Faculty of Social Work, University of Toronto, Toronto, Canada; 10 Division of Adolescent and Young Adult Medicine, University of California, San Francisco, 550 16th Street, #4206, San Francisco, CA 94158 USA

**Keywords:** Firearm storage, Neighborhood safety, Crime, Injury prevention, Behavioral risk factor surveillance system

## Abstract

**Background:**

Secure firearm storage (i.e., keeping firearms locked and ammunition stored separately) is a means of reducing risks of suicide, unintentional injury, and homicide. While prior research has examined demographic and household factors associated with storage practices, less is known about how contextual factors, such as perceived neighborhood safety from crime, influence firearm storage behavior.

**Methods:**

Data were from the 2024 Behavioral Risk Factor Surveillance System (BRFSS), a nationally coordinated, state-based survey administered by state health departments with technical and methodological support from the Centers for Disease Control and Prevention. Analyses were restricted to respondents in firearm-owning households from ten states (*n* = 18,443). Firearm storage was categorized as: (1) unloaded [safest], (2) loaded and locked [intermediate risk], or (3) loaded and unlocked [unsafe storage practice]. Perceived neighborhood safety from crime was classified as extremely safe, safe, or unsafe (unsafe and extremely unsafe combined). Multinomial logistic regression models were used to assess the associations between perceived neighborhood safety and firearm storage.

**Results:**

Most respondents lived in households with firearms stored unloaded (63.2%), followed by loaded and locked (19.8%) and loaded and unlocked (17.0%). Compared with those who perceived their neighborhoods as extremely safe, respondents who perceived them as unsafe or extremely unsafe had a higher relative risk of living in a household with firearms loaded and unlocked (RRR = 1.773; 95% CI: 1.240–2.536).

**Conclusions:**

Firearm injury prevention initiatives addressing storage practices should consider the role of community-level perceptions of safety through intervention-oriented and policy-relevant approaches, including via the integration of public health and violence prevention strategies.

## Introduction

As of 2024, an estimated 48% of Americans reported having a firearm in their household [1]. Self-defense and personal safety are commonly cited as primary reasons for firearm ownership [2]. Yet, the presence of firearms in the home is associated with elevated risks of suicide, unintentional injury, and homicide [3–6]. Secure firearm storage (i.e., keeping firearms locked and storing ammunition separately) is a strategy for mitigating these risks [7, 8]. Even so, an estimated 25–40% of firearm-owning households store at least one firearm in an unsecured location [9]. Although prior studies have examined individual- and household-level correlates of firearm storage behavior [10, 11, 12, 13, 14], less is known about how community factors, such as perceived neighborhood safety from crime, influence storage practices. Individuals who perceive their neighborhoods as unsafe may be more likely to store firearms unlocked to enable rapid access in the event of a threat [10, 11, 15]. The current study uses new data from ten states participating in the 2024 Behavioral Risk Factor Surveillance System to examine the association between perceived neighborhood safety and firearm storage behavior. We hypothesize that firearm owners who perceive their neighborhoods as less safe will be more likely to be in households where firearms are stored unlocked and loaded compared with those who perceive their neighborhoods as safe.

## Methods

Data for this study come from the 2024 BRFSS, a nationally coordinated, state-based telephone survey administered by state health departments with technical and methodological support from the Centers for Disease Control and Prevention. The BRFSS collects information from adults aged 18 years and older across the United States to monitor a wide range of health behaviors and risk factors. Although the BRFSS is conducted in all 50 states and Washington, DC, some states opt to administer additional modules on specific topics. This study draws on data from 10 states that included the optional module on firearm safety and were asked state-optional questions on neighborhood safety (California, Indiana, Louisiana, Minnesota, Mississippi, New Jersey, New Mexico, North Carolina, South Carolina, West Virginia). Details on sample selection procedures and the states included in the sample are provided in Appendices A and B. Because the BRFSS data are publicly available and fully deidentified, this study was exempt from Institutional Review Board review.

### Measures

*Perceived neighborhood safety* was based on the following question: “How safe from crime do you consider your neighborhood to be?” (extremely safe, safe, unsafe, or extremely unsafe). Consistent with prior research [14, 16], *firearm storage* was measured as follows: First, respondents who reported living in a household with any firearms kept in or around the home (excluding firearms that cannot fire) were asked a series of questions about how those firearms were stored. First, respondents were asked, “Are any of these firearms now loaded?” (yes or no). Those who reported living in households with firearms stored while loaded were then asked, “Are any of these loaded firearms also unlocked?” (yes or no). Using these questions, we classify respondents into the following categories: (1) unloaded [safest], (2) loaded and locked [intermediate risk], or (3) loaded and unlocked [unsafe storage practice] [14]. 

### Control variables

Models adjusted for demographic, socioeconomic, health, and contextual covariates that prior research has shown to be associated with firearm ownership and storage practices and that may confound the relationship between perceived neighborhood safety and firearm storage behavior. Demographic characteristics included age (18–24, 25–34, 35–44, 45–54, 55–64, or ≥ 65 years), sex (male or female), and race/ethnicity (non-Hispanic White, non-Hispanic Black, non-Hispanic Multiracial, non-Hispanic other race, or Hispanic), as firearm ownership patterns, perceived risk, and storage behaviors vary systematically across demographic characteristics [10, 14, 17, 18]. Marital status (married; divorced or separated; widowed; never married; or member of an unmarried couple) was included to account for household composition and social context, which may influence both safety perceptions and household storage decisions [14, 18]. 

Socioeconomic status was captured through educational attainment (less than high school, high school graduate, some college, or college graduate) and household income (<$25,000; $25,000–$49,999; $50,000–$74,999; $75,000–$99,999; $100,000–$149,999; or ≥$150,000), given evidence that economic resources shape access to secure storage devices and broader risk-management strategies [14, 19, 20]. Additional household-level factors included whether a child lived in the home (yes or no), which serves as a consistently strong predictor of safer storage practices [9, 10, 14, 17, 18], and veteran status (yes or no), as veterans have higher rates of firearm ownership and may differ in firearm training, perceived threat, and storage behaviors [21–23]. 

To more fully capture economic and material hardship beyond income alone, social determinants of health were measured using six indicators reflecting respondents’ experiences in the past 12 months: job loss, receipt of Supplemental Nutrition Assistance Program (SNAP) benefits, food insecurity, inability to pay mortgage, rent, or utility bills, threats of utility shutoff, and unreliable transportation (yes or no). Affirmative responses were summed and categorized as 0, 1, 2, or ≥ 3 indicators, reflecting cumulative exposure to material hardship that may influence firearm storage practices [14, 19]. Finally, models adjusted for self-rated health (poor/fair vs. good/very good/excellent) and lifetime depression diagnosis (yes or no) to account for health factors that may affect threat perception, hypervigilance, and risk-taking behaviors [14, 24]. Urbanicity (urban vs. rural) and state of residence were included to account for geographic variation in crime exposure, firearm norms, policy environments, and storage behaviors [10, 14, 25]. 

### Analytic approach

We first computed unweighted counts and weighted proportions for all study variables. To examine associations between perceived neighborhood safety and firearm storage practices, we estimated multinomial logistic regression models. All analyses accounted for the complex sampling design of the BRFSS by applying survey weights using the *svy* commands in Stata version 18 (StataCorp). Statistical significance was defined as *p* < 0.05.

## Results

Among the 18,443 respondents in firearm-owning households, 63.2% reported living in households with firearms stored unloaded and locked, 19.8% lived in households with firearms stored loaded and locked, and 17.0% with firearms stored loaded and unlocked. In terms of perceived neighborhood safety, 38.2% described their neighborhood as extremely safe from crime, 56.8% as safe, 4.3% as unsafe, and 0.8% as extremely unsafe (see Table 1). Because few respondents reported feeling extremely unsafe, the “unsafe” and “extremely unsafe” categories were combined for regression analyses. Summary statistics stratified by firearm secure storage status are provided in Appendix C.

**Table 1:** Weighted summary statistics of the analytic sample from the behavioral risk factor surveillance System, 2024 (*N* = 18,443)

Variable	Weighted % (Unweighted *n*)	95% Confidence Interval (%)
*Firearm Storage*		
Unloaded	63.2% (11,823)	61.9–64.6
Loaded & Locked	19.8% (3,158)	18.6–20.9
Loaded & Unlocked	17.0% (3,462)	16.0–18.0
*Perceived Neighborhood Safety*		
Extremely Safe	38.2% (7,732)	36.9–39.6
Safe	56.8% (9,983)	55.4–58.2
Unsafe	4.2% (628)	3.8–4.8
Extremely Unsafe	0.8% (100)	0.5–1.0
*Age*		
18–24	7.5% (703)	6.6–8.5
25–34	14.1% (1,619)	13.0–15.2
35–44	16.6% (2,394)	15.4–17.8
45–54	17.6% (2,953)	16.4–18.7
55–64	18.8% (3,820)	17.7–19.8
65+	25.4% (6,954)	24.3–26.6
*Sex*		
Female	42.5% (8,295)	41.1–43.9
Male	57.5% (10,148)	56.1–58.9
*Race/Ethnicity*		
Non-Hispanic White	70.8% (15,078)	69.3–72.3
Non-Hispanic Black	11.6% (1,721)	10.7–12.5
Non-Hispanic Other	5.7% (510)	4.6–6.8
Non-Hispanic Multiracial	2.0% (278)	1.6–2.5
Hispanic	9.9% (856)	8.7–11.1
*Marital Status*		
Married	61.0% (11,442)	59.6–62.4
Divorced/Separated	12.0% (2,421)	11.1–12.9
Widowed	6.1% (1,705)	5.6–6.7
Never Married	16.0% (2,160)	14.9–17.2
Member of an Unmarried Couple	4.8% (715)	4.2–5.4
*Educational Attainment*		
Less than High School	5.8% (617)	5.1–6.5
High School Graduate	27.3% (4,569)	26.0–28.6
Some College	34.8% (5,355)	33.3–36.2
College Graduate	32.2% (7,902)	30.9–33.4
*Household Income*		
Less than $25,000	6.9% (1,462)	6.3–7.5
$25,000 - $49,999	20.0% (4,079)	18.9–21.1
$50,000 - $74,999	16.7% (3,230)	15.6–17.8
$75,000 - $99,999	16.8% (3,028)	15.6–18.0
$100,000 - $149,999	18.3% (3,368)	17.2–19.3
$150,000 or more	21.3% (3,276)	20.1–22.6
*Child in Home*		
None	66.6% (13,723)	65.2–68.0
1 or more	33.4% (4,720)	32.0–34.8
*Veteran Status*		
No	85.7% (15,560)	84.8–86.7
Yes	14.3% (2,883)	13.3–15.2
*Household Income*		
Less than $25,000	6.9% (1,462)	6.3–7.5
$25,000 - $49,999	20.0% (4,079)	18.9–21.1
$50,000 - $74,999	16.7% (3,230)	15.6–17.8
$75,000 - $99,999	16.8% (3,028)	15.6–18.0
$100,000 - $149,999	18.3% (3,368)	17.2–19.3
$150,000 or more	21.3% (3,276)	20.1–22.6
*Social Determinants of Health*		
0	76.6% (14,809)	75.3–77.9
1	14.1% (2,130)	12.9–15.3
2	5.0% (811)	4.4–4.7
3	4.3% (693)	3.7–4.8
*Self-Rated Health*		
Excellent, Very Good, or Good	83.5% (15,166)	82.4–84.6
Fair or Poor	16.5% (3,277)	15.4–17.6
*Depression*		
No	78.5% (14,526)	77.3–79.7
Yes	21.5% (3,917)	20.3–22.7
*Urbanicity*		
Rural	9.5% (2,653)	8.9–10.0
Urban	90.5% (15,790)	90.0–91.1

Multivariable multinomial logistic regression results reported in Table 2 indicated that, relative to respondents in households with firearms stored unloaded, those living in neighborhoods perceived as unsafe had a significantly higher relative risk of being in households storing firearms loaded and unlocked (relative risk ratio [RRR] = 1.773; 95% Confidence Interval [CI]: 1.240–2.536) compared with those living in neighborhoods perceived as extremely safe. When retaining the original four-category safety measure, the risk of storing firearms loaded and unlocked was higher among those reporting neighborhoods as unsafe (RRR = 1.576; 95% CI: 1.078–2.304) and extremely unsafe (RRR = 3.253; 95% CI: 1.433–7.386) relative to those reporting extremely safe neighborhoods (see Appendix D). Finally, analyses using multiplicative interaction terms revealed that the relationship between perceived neighborhood safety and firearm storage did not differ significantly across demographic characteristics, including age, sex, race/ethnicity, and whether a child was present in the home (results available upon request).

**Table 2:** Multinomial logistic regression of firearm storage on neighborhood safety (*N* = 18,443)

Variable	Loaded & Locked vs. Unloaded		Loaded & Unlocked vs. Unloaded	
	RRR	95% CI	RRR (95% CI)	95% CI
*Perceived Neighborhood Safety* (Ref = Extremely Safe)	—	—	—	—
Safe	0.987	(0.836–1.164)	0.934	(0.802–1.089)
Unsafe or Extremely Unsafe	1.086	(0.736–1.603)	1.773**	(1.240–2.536)
*Age* (Ref = 18–24)	—	—	—	—
25–34	1.198	(0.825–1.738)	1.684*	(1.126–2.519)
35–44	1.227	(0.826–1.821)	1.838**	(1.225–2.758)
45–54	1.186	(0.803–1.753)	2.118***	(1.399–3.205)
55–64	1.163	(0.777–1.741)	1.721**	(1.152–2.572)
65+	0.897	(0.595–1.352)	1.516*	(1.013–2.271)
*Sex* (Ref = Female)	—	—	—	—
Male	1.521***	(1.284–1.802)	2.216***	(1.876–2.618)
*Race/Ethnicity* (Ref = Male)	—	—	—	—
Non-Hispanic Black	1.411**	(1.092–1.822)	0.963	(0.747–1.242)
Non-Hispanic Other	1.058	(0.630–1.777)	0.842	(0.530–1.340)
Non-Hispanic Multiracial	1.151	(0.631–2.099)	1.549	(0.932–2.574)
Hispanic	0.847	(0.593–1.209)	1.041	(0.678–1.600)
*Marital Status* (Ref = Married)	—	—	—	—
Divorced/Separated	1.023	(0.801–1.306)	1.469***	(1.174–1.838)
Widowed	1.004	(0.747–1.349)	1.838***	(1.391–2.428)
Never Married	0.883	(0.677–1.151)	1.184	(0.897–1.563)
Member of an Unmarried Couple	0.919	(0.629–1.344)	1.373	(0.983–1.919)
*Educational Attainment* (Ref = Less than High School)	—	—	—	—
High School Graduate	1.381	(0.926–2.057)	1.277	(0.874–1.865)
Some College	1.203	(0.811–1.783)	1.265	(0.858–1.866)
College Graduate	0.962	(0.645–1.434)	0.911	(0.607–1.366)
*Child in Home* (Ref = None)	—	—	—	—
1 or more	0.908	(0.746–1.107)	0.342***	(0.275–0.426)
*Veteran Status* (Ref = No)	—	—	—	—
Yes	1.068	(0.856–1.331)	1.158	(0.946–1.418)
*Household Income* (Ref = <$25,000)	—	—	—	—
$25,000 - $49,999	1.111	(0.829–1.490)	0.795	(0.600–1.055)
$50,000 - $74,999	1.363	(0.968–1.918)	1.153	(0.839–1.584)
$75,000 - $99,999	1.065	(0.760–1.493)	1.095	(0.792–1.513)
$100,000 - $149,999	1.500*	(1.061–2.121)	1.306	(0.932–1.831)
$150,000 or more	1.499*	(1.054–2.132)	1.284	(0.888–1.857)
*Social Determinants of Health* (Ref = 0)	—	—	—	—
1	1.068	(0.832–1.372)	0.964	(0.764–1.217)
2	1.173	(0.850–1.619)	0.824	(0.593–1.145)
3	1.263	(0.906–1.762)	0.960	(0.674–1.368)
*General Health* (Ref = Excellent/Very Good/Good)	—	—	—	—
Fair or Poor	1.190	(0.943–1.502)	1.275*	(1.030–1.578)
*Depression* (Ref = No)	—	—	—	—
Yes	0.796*	(0.644–0.983)	0.916	(0.759–1.105)
*Urbanicity* (Ref = Rural)	—	—	—	—
Urban	1.105	(0.901–1.355)	0.959	(0.805–1.143)

*** *p* < 0.001, ** *p* < 0.01, * *p* < 0.05

RRR = relative risk ratio; CI = confidence interval

Control variables for state of residence not shown

## Discussion

Using data from more than 18,000 US adults residing in households with firearms, the current study finds that feeling one’s neighborhood is unsafe is significantly associated with a higher likelihood of being in a household with firearms stored loaded and unlocked. The findings are consistent with recent research showing that perceived neighborhood safety is a factor associated with firearm storage behavior [10, 11, 15]. The findings can also be understood within the context of existing theories, suggesting that feeling unsafe and experiencing heightened hypervigilance can contribute to behaviors that favor short-term survival strategies, such as storing a loaded and unlocked firearm in the household [11, 15, 26]. 

The findings of this study demonstrate the importance of addressing perceived neighborhood safety as part of firearm injury prevention efforts. Public health interventions and educational campaigns that promote secure firearm storage often focus on individual- and household-level factors but rarely consider the broader social context in which storage decisions are made [7, 8]. The current results suggest that feeling unsafe in their neighborhoods may contribute to unsecured firearm storage practices. Interventions that promote secure firearm storage may be more effective if they explicitly acknowledge and address perceptions of neighborhood safety that motivate individuals to prioritize rapid access to firearms. Clinicians and community-based programs could incorporate brief, trauma- and safety-informed counseling that validates concerns about personal security while emphasizing strategies to maintain both readiness and safety, such as quick-access lock technology [7, 13, 27, 28]. 

At the policy level, the results highlight the importance of pairing household-level firearm safety initiatives with broader investments in community violence prevention, neighborhood stabilization, and crime reduction efforts that may indirectly reduce reliance on unsafe storage practices. Integrating secure storage promotion into place-based public health strategies, such as community violence intervention programs, housing and neighborhood revitalization initiatives, and public safety partnerships, may yield greater and more sustained reductions in firearm-related injury risk. For instance, a prior study demonstrated the feasibility of distributing firearm storage devices at a community summit focused on reducing violence [29]. Whether and how to best integrate firearm storage counseling and device distribution into other community violence reduction initiatives is an important area of future research.

### Limitations and future directions

The current study has limitations that can be expanded upon in future research. First, firearm storage and neighborhood safety questions were included in only 10 states in the 2024 BRFSS, which limits the generalizability of the findings to the broader US population. Second, because of the potential for unmeasured confounding, the reported relationships should be interpreted as associations rather than causal effects. Third, the cross-sectional nature of the BRFSS prevents assessment of temporal ordering, making it impossible to determine whether changes in perceived neighborhood safety over time correspond with changes in firearm storage behavior. Fourth, due to the wording of the firearm storage question in the BRFSS, the reference category is storing a firearm unloaded. However, those respondents were not also asked if a firearm was stored in a locked location.

## Conclusions

The current study contributes to a growing body of research suggesting that perceptions of community safety from crime are a key factor in household firearm storage behavior. Future research and data collection are needed, including longitudinal studies and mixed-methods approaches, to determine why perceptions of safety influence firearm storage.

## Data Availability

The Behavioral Risk Factor Surveillance Data used in this study are publicly available at: https://www.cdc.gov/brfss/index.html. All replication code is available from the corresponding author upon reasonable request.

## Appendix A: sample selection flowchart

**Table:**

Participated in the 2024 BRFSS	N = 457,670
	↓
Participated in the “Firearm Safety” Module	N = 99,396
	↓
Lives in a Firearm Owning Household	N = 38,004
	↓
Answered Question on Firearm Storage	N = 36,847
	↓
Answered Questions on Neighborhood Crime and Safety	N = 21,491
	↓
Has Data on Control Variables	N = 18,443

## Appendix B: respondents per state (*N* = 18,443)

**Table:**

	Number of Respondents
California	831
Indiana	3,442
Louisiana	1,497
Minnesota	3,705
Mississippi	1,245
New Jersey	762
New Mexico	866
North Carolina	927
South Carolina	2,686
West Virginia	2,482

## Appendix C: weighted summary statistics of the analytic sample stratified by firearm storage status

**Table:**

Variable	Unloaded (*N* = 11,823)	Loaded & Locked (*N* = 3,158)	Loaded & Unlocked (*N* = 3,462)	
	Weighted % [95% CI]	Weighted % [95% CI]	Weighted % [ 95% CI]	*p*-value
**Perceived Neighborhood Safety**				< 0.001
Extremely Safe	38.4% [36.6–40.2]	37.7% [34.4–40.9]	38.2% [35.5–41.0]	
Safe	57.1% [55.3–59.0]	57.5% [54.2–60.8]	54.7% [51.7–57.7]	
Unsafe	4.0% [3.1–4.8]	3.8% [2.7–5.0]	5.5% [4.2–6.8]	
Extremely Unsafe	0.5% [0.2–0.8]	1.0% [0.5–1.5]	1.5% [0.6–2.4]	
**Age**				< 0.001
18–24	8.2% [6.9–9.4]	7.3% [5.4–9.1]	5.6% [4.2–7.0]	
25–34	14.2% [12.8–15.7]	15.2% [13.0–17.4]	12.5% [10.4–14.5]	
35–44	17.3% [15.8–18.8]	18.3% [15.2–21.5]	12.0% [10.1–14.0]	
45–54	16.8% [15.4–18.2]	18.5% [16.0–20.9]	19.4% [16.4–23.0]	
55–64	17.9% [16.7–19.2]	19.4% [16.6–22.2]	21.0% [18.7–23.3]	
65+	25.6% [24.2–27.1]	21.3% [18.9–23.7]	29.5% [27.0–32.0]	
**Sex**				< 0.001
Female	47.7% [45.8–49.6]	37.0% [33.9–40.0]	29.5% [26.8–32.2]	
Male	52.3% [50.4–54.2]	63.0% [59.9–66.1]	70.5% [68.9–73.2]	
**Race/Ethnicity**				< 0.001
Non-Hispanic White	71.0% [69.0–73.0]	67.6% [64.2–71.0]	73.8% [70.6–77.0]	
Non-Hispanic Black	9.9% [8.9–10.8]	16.1% [13.5–18.6]	12.8% [10.6–14.9]	
Non-Hispanic Other	6.3% [4.8–7.7]	5.7% [3.0–8.3]	3.5% [2.3–4.7]	
Non-Hispanic Multiracial	1.9% [1.3–2.4]	2.1% [1.0–3.1]	2.6% [1.6–3.5]	
Hispanic	11.0% [9.4–12.7]	8.6% [6.4–10.8]	7.3% [4.8–9.9]	
**Marital Status**				< 0.001
Married	62.0% [60.1–63.8]	63.4% [60.3–66.5]	54.6% [51.6–57.6]	
Divorced/Separated	11.1% [10.0–12.3]	11.6% [9.7–13.5]	15.6% [13.6–17.6]	
Widowed	5.8% [5.1–6.6]	4.8% [3.7–5.9]	8.9% [7.4–10.3]	
Never Married	16.2% [14.7–17.8]	15.7% [13.3–18.0]	15.8% [13.5–18.1]	
Unmarried Couple	4.8% [4.0–5.6]	4.5% [3.2–5.8]	5.1% [3.9–6.3]	
**Educational Attainment**				< 0.001
Less than High School	5.5% [4.7–6.4]	5.5% [3.9–7.1]	6.9% [5.2–8.5]	
High School Graduate	25.7% [24.0–27.4]	30.4% [27.2–33.7]	29.6% [26.9–32.3]	
Some College	34.2% [32.4–36.1]	34.6% [31.3–37.9]	36.9% [33.9–40.0]	
College Graduate	34.5% [32.8–36.1]	29.5% [26.7–32.3]	26.6% [24.2–28.9]	
**Child in Home**				< 0.001
None	63.8% [61.9–65.7]	62.0% [58.7–65.3]	82.4% [80.1–84.7]	
1 or more	36.2% [34.3–38.1]	38.0% [34.7–41.3]	17.6% [15.3–19.9]	
**Veteran Status**				< 0.001
No	87.4% [86.1–88.7]	84.7% [82.5–86.9]	80.8% [78.7–82.9]	
Yes	12.6% [11.3–13.9]	15.3% [13.1–17.5]	19.2% [17.1–21.3]	
**Household Income**				< 0.001
Less than $25,000	6.5% [5.7–7.3]	6.1% [5.0–7.3]	9.3% [7.8–10.9]	
$25,000–$49,999	20.3% [18.9–21.7]	19.5% [17.2–21.9]	19.4% [17.3–21.5]	
$50,000–$74,999	16.2% [14.8–17.6]	17.6% [14.7–20.5]	17.7% [15.1–20.2]	
$75,000–$99,999	17.3% [15.8–18.9]	14.5% [12.2–16.8]	17.5% [15.2–19.8]	
$100,000–$149,999	17.7% [16.5–19.0]	20.0% [17.2–22.8]	18.1% [16.0–20.3]	
$150,000 or more	22.0% [20.3–23.6]	22.3% [19.4–25.1]	18.0% [15.3–20.6]	
**Social Determinants of Health**				< 0.001
0	76.7% [74.9–78.4]	74.9% [71.9–77.9]	78.3% [76.0–80.6]	
1	14.2% [12.6–15.8]	14.6% [11.8–17.3]	13.2% [11.3–15.2]	
2	4.9% [4.1–5.8]	5.8% [4.4–7.2]	4.5% [3.5–5.5]	
3 or more	4.2% [3.4–5.0]	4.7% [3.6–5.8]	4.0% [3.0–5.0]	
**Self-Rated Health**				< 0.001
Excellent/Very Good/Good	85.0% [83.8–86.2]	82.7% [79.9–85.6]	78.9% [76.3–81.5]	
Fair/Poor	15.0% [13.8–16.2]	17.3% [14.4–20.1]	21.1% [18.5–23.7]	
**Depression**				0.217
No	77.4% [75.8–78.9]	81.1% [78.5–83.7]	79.7% [77.3–82.0]	
Yes	22.6% [21.1–24.2]	18.9% [16.3–21.5]	20.3% [18.0–22.7]	
**Urbanicity**				0.005
Rural	9.1% [8.3–9.8]	8.9% [7.7–10.2]	11.5% [10.2–12.8]	
Urban	90.9% [90.2–91.7]	91.1% [89.8–92.3]	88.5% [87.2–89.8]	

* p*-value reflects results from chi-squared tests

## Appendix D: multinomial logistic regression of firearm storage on levels of neighborhood safety (*N* = 18,443)

**Table:**

Variable	Loaded & Locked vs. Unloaded		Loaded & Unlocked vs. Unloaded	
	RRR	95% CI	RRR (95% CI)	95% CI
*Perceived Neighborhood Safety* (Ref = Extremely Safe)	—	—	—	—
Safe	0.988	(0.838–1.166)	0.936	(0.804–1.090)
Unsafe	0.975	(0.631–1.505)	1.576*	(1.078–2.304)
Extremely Unsafe	1.960	(0.952–4.039)	3.253**	(1.433–7.386)

*** *p* < 0.001, ** *p* < 0.01, * *p* < 0.05

RRR = relative risk ratio; CI = confidence interval

Control variables include age, sex, race/ethnicity, marital status, educational attainment, child in home, veteran status, household income, social determinants of health, self-reported health, depression, urbanicity, and state of residence
